# Oxido{*N*-[(2-oxido-1-naphthyl-κ*O*)methyl­idene]asparaginato-κ^2^ *O* ^1^,*N* ^2^}(1,10-phenanthroline-κ^2^ *N*,*N*′)vanadium(IV) *N*,*N*-dimethyl­formamide monosolvate

**DOI:** 10.1107/S1600536810030126

**Published:** 2010-08-04

**Authors:** Lin Bian, Lianzhi Li, Qingfu Zhang, Daqi Wang

**Affiliations:** a School of Chemistry and Chemical Engineering, Liaocheng University, Shandong 252059, People’s Republic of China

## Abstract

The tridentate Schiff base ligand of the title complex, [V(C_15_H_12_N_2_O_4_)O(C_12_H_8_N_2_)]·C_3_H_7_NO, was derived from the condensation of 2-hy­droxy-1-naphthaldehyde and l-asparagine. The central V^IV^ atom is six-coordinated by one oxide O atom, two N atoms from 1,10-phenanthroline and one N atom and two O atoms from the Schiff base ligand in a distorted octa­hedral geometry. In the crystal structure, inter­molecular N—H⋯O hydrogen bonds connect mol­ecules into centrosymmetric dimers. The C atoms of the dimethyl­formamide solvent mol­ecule are disordered over two sites with site-occupancy factors of 0.732 (13) and 0.268 (13).

## Related literature

For the insulin-mimetic properties of vanadium compounds, see: Diego *et al.* (2003 ▶); Kenji *et al.* (2000 ▶); Thompson & Orvig (2006 ▶). For related structures, see: Hoshina *et al.* (1998 ▶); Otieno *et al.* (1996 ▶).
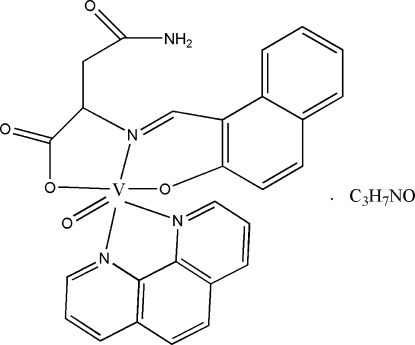


## Experimental

### 

#### Crystal data

[V(C_15_H_12_N_2_O_4_)O(C_12_H_8_N_2_)]·C_3_H_7_NO
*M* *_r_* = 604.51
Triclinic, 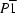

*a* = 10.357 (1) Å
*b* = 11.1021 (12) Å
*c* = 12.9119 (14) Å
α = 101.396 (2)°
β = 104.196 (2)°
γ = 91.010 (1)°
*V* = 1407.5 (3) Å^3^
*Z* = 2
Mo *K*α radiation
μ = 0.41 mm^−1^
*T* = 298 K
0.36 × 0.31 × 0.25 mm

#### Data collection

Bruker SMART 1000 CCD area-detector diffractometer
Absorption correction: multi-scan (*SADABS*; Sheldrick, 1996 ▶) *T*_min_ = 0.868, *T*_max_ = 0.905
7387 measured reflections
4892 independent reflections
3123 reflections with *I* > 2σ(*I*)
*R*_int_ = 0.063

#### Refinement

*R*[*F* ^2^ > 2σ(*F* ^2^)] = 0.075
*wR*(*F* ^2^) = 0.216
*S* = 1.00
4892 reflections
411 parameters
H-atom parameters constrained
Δρ_max_ = 0.74 e Å^−3^
Δρ_min_ = −0.40 e Å^−3^

### 

Data collection: *SMART* (Bruker, 1996 ▶); cell refinement: *SAINT* (Bruker, 1996 ▶); data reduction: *SAINT*; program(s) used to solve structure: *SHELXS97* (Sheldrick, 2008 ▶); program(s) used to refine structure: *SHELXL97* (Sheldrick, 2008 ▶); molecular graphics: *SHELXTL* (Sheldrick, 2008 ▶); software used to prepare material for publication: *SHELXTL*.

## Supplementary Material

Crystal structure: contains datablocks global, I. DOI: 10.1107/S1600536810030126/pv2309sup1.cif

Structure factors: contains datablocks I. DOI: 10.1107/S1600536810030126/pv2309Isup2.hkl

Additional supplementary materials:  crystallographic information; 3D view; checkCIF report

## Figures and Tables

**Table 1:** Hydrogen-bond geometry (Å, °)

*D*—H⋯*A*	*D*—H	H⋯*A*	*D*⋯*A*	*D*—H⋯*A*
N1—H1*A*⋯O3^i^	0.86	2.05	2.909 (5)	175
N1—H1*B*⋯O6^ii^	0.86	2.00	2.852 (6)	169

Symmetry codes: (i) 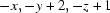; (ii) 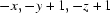.

**Fig. 1.**
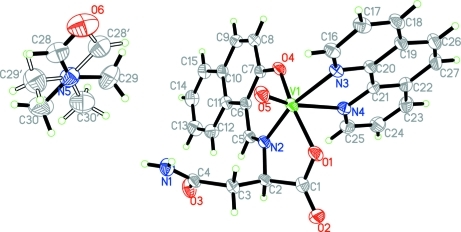
The molecular structure of the title compound, showing 30% probability displacement ellipsoids and the atom-numbering scheme. The smaller fraction of the disordered C-atoms of the solvate have been joined by hollow bonds.

**Fig. 2.**
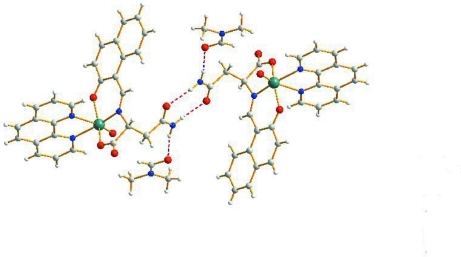
Hydrogen bonding interactions in the title compound shown as dashed lines.

## supplementary crystallographic information

### Comment

Vanadium is a biologically essential trace element, encountered in metalloenzymes such as haloperoxidases or nitrogenases. The coordination chemistry of oxovanadium (IV) has gained a great interest due to the fact that vanadium compounds in various oxidation states have insulin-mimetic properties (Kenji, *et al.*, 2000; Diego *et al.*, 2003; Thompson & Orvig, 2006). We report here the synthesis and crystal structure of the title complex.

In the molecular structure of the title compound (Fig. 1), the tridentate Schiff base ligand is derived from the condensation of 2-hydroxy-1-naphthaldehyde and L-asparagine. The central V^IV^ atom is six-coordinated by one oxide O atom, two N atoms from 1,10-phenanthroline and one N atom and two O atoms from the schiff base ligand in a distorted octahedral geometry. The V═O bond distance is 1.587 (3)Å which is typical for oxovandium complexes (Hoshina *et al.*, 1998; Otieno *et al.*, 1996). The Schiff base with the vanadium atom has formed a five-member ring (O1/C1–2/N2/V1) and a six-member ring (N2/C5–7/O4/V1], and the two rings have the dihedral angle 20.89 (17)°, which increases the stability of the complex. Furthermore, the 1,10–phenanthroline ligand is almost perpendicular to the equatorial plane [dihedral angle 84.98 (8)°].

In the crystal structure, the intermolecular N—H···O hydrogen bonds (Table 1) connect molecules into centrosymmetric dimers (Fig. 2). The solvate molecules are also hydrogen bonded to the Schiff base ligand. The structure is stablilized by inter- and intra-molecular hydrogen bonds of the type C—H···O.

### Experimental

L-Asparagine (1 mmol, 150.1 mg) and potassium hydroxide (1 mmol, 56.1 mg) were dissolved in hot methanol (5 ml) with stirring and added successively to a methanol solution (5 ml) of 2-hydroxy-1-naphthaldehyde (1 mmol, 172.2 mg). The mixture was stirred at 323 K for 2 h. Subsequently, an aqueous solution (2 ml) of vanadyl sulfate hydrate (1 mmol, 225.4 mg) was added dropwise and stirred for 2 h continuously. 1,10-Phenanthroline (1 mmol, 198.2 mg) was then added to the stirring mixture and further refluxed for 4 h and then filtered. The precipitate was dissolved in *N*,*N*-dimethylformamide (10 ml) and held at room temperature for several days, whereupon brown blocky crystals suitable for X-ray diffraction were obtained.

### Refinement

All H atoms were placed in geometrically calculated positions, with C—H = 0.93–0.98 Å, and allowed to ride on their respective parent atoms, with *U*_iso_(H) = 1.2*U*_eq_(C) or 1.5*U*_eq_(C_methyl_). The C-atoms of the *N*,*N*-dimethylformamide solvate were disordered over two sites with site occupancy factors 0.732 (13) and 0.268 (13).

### Crystal data

**Table:**

[V(C_15_H_12_N_2_O_4_)O(C_12_H_8_N_2_)]·C_3_H_7_NO	*Z* = 2
*M**_r_* = 604.51	*F*(000) = 626
Triclinic, *P*1	*D*_x_ = 1.426 Mg m^−^^3^
Hall symbol: -P 1	Mo *K*α radiation, λ = 0.71073 Å
*a* = 10.357 (1) Å	Cell parameters from 1024 reflections
*b* = 11.1021 (12) Å	θ = 2.2–24.7°
*c* = 12.9119 (14) Å	µ = 0.41 mm^−^^1^
α = 101.396 (2)°	*T* = 298 K
β = 104.196 (2)°	Block, brown
γ = 91.010 (1)°	0.36 × 0.31 × 0.25 mm
*V* = 1407.5 (3) Å^3^	

### Data collection

**Table:**

Bruker SMART 1000 CCD area-detector diffractometer	4892 independent reflections
Radiation source: fine-focus sealed tube	3123 reflections with *I* > 2σ(*I*)
graphite	*R*_int_ = 0.063
φ and ω scans	θ_max_ = 25.0°, θ_min_ = 1.7°
Absorption correction: multi-scan (*SADABS*; Sheldrick, 1996)	*h* = −9→12
*T*_min_ = 0.868, *T*_max_ = 0.905	*k* = −13→13
7387 measured reflections	*l* = −13→15

### Refinement

**Table:**

Refinement on *F*^2^	Primary atom site location: structure-invariant direct methods
Least-squares matrix: full	Secondary atom site location: difference Fourier map
*R*[*F*^2^ > 2σ(*F*^2^)] = 0.075	Hydrogen site location: inferred from neighbouring sites
*wR*(*F*^2^) = 0.216	H-atom parameters constrained
*S* = 1.00	*w* = 1/[σ^2^(*F*_o_^2^) + (0.13*P*)^2^] where *P* = (*F*_o_^2^ + 2*F*_c_^2^)/3
4892 reflections	(Δ/σ)_max_ = 0.001
411 parameters	Δρ_max_ = 0.74 e Å^−^^3^
0 restraints	Δρ_min_ = −0.40 e Å^−^^3^

### Special details

**Table:**

Geometry. All e.s.d.'s (except the e.s.d. in the dihedral angle between two l.s. planes) are estimated using the full covariance matrix. The cell e.s.d.'s are taken into account individually in the estimation of e.s.d.'s in distances, angles and torsion angles; correlations between e.s.d.'s in cell parameters are only used when they are defined by crystal symmetry. An approximate (isotropic) treatment of cell e.s.d.'s is used for estimating e.s.d.'s involving l.s. planes.
Refinement. Refinement of *F*^2^ against ALL reflections. The weighted *R*-factor *wR* and goodness of fit *S* are based on *F*^2^, conventional *R*-factors *R* are based on *F*, with *F* set to zero for negative *F*^2^. The threshold expression of *F*^2^ > σ(*F*^2^) is used only for calculating *R*-factors(gt) *etc*. and is not relevant to the choice of reflections for refinement. *R*-factors based on *F*^2^ are statistically about twice as large as those based on *F*, and *R*- factors based on ALL data will be even larger.

### Fractional atomic coordinates and isotropic or equivalent isotropic displacement parameters (Å^2^)

**Table:**

	*x*	*y*	*z*	*U*_iso_*/*U*_eq_	Occ. (<1)
V1	0.21794 (8)	0.72939 (7)	0.12980 (6)	0.0417 (3)	
N1	−0.0395 (4)	0.8528 (4)	0.4060 (3)	0.0547 (11)	
H1A	−0.0235	0.8842	0.4747	0.066*	
H1B	−0.0620	0.7755	0.3824	0.066*	
N2	0.1611 (3)	0.9001 (3)	0.1906 (3)	0.0381 (8)	
N3	0.2777 (3)	0.5734 (3)	0.0261 (3)	0.0402 (9)	
N4	0.2988 (4)	0.8051 (3)	−0.0030 (3)	0.0435 (9)	
N5	0.0667 (5)	0.5958 (5)	0.6803 (4)	0.0717 (13)	
O1	0.0481 (3)	0.7362 (3)	0.0169 (3)	0.0495 (8)	
O2	−0.1224 (3)	0.8505 (3)	−0.0363 (3)	0.0579 (9)	
O3	0.0023 (4)	1.0347 (3)	0.3643 (3)	0.0595 (10)	
O4	0.4011 (3)	0.7907 (3)	0.2145 (2)	0.0446 (8)	
O5	0.1758 (3)	0.6444 (3)	0.2031 (3)	0.0547 (9)	
O6	0.1289 (5)	0.4015 (4)	0.6468 (5)	0.1162 (19)	
C1	−0.0249 (5)	0.8286 (4)	0.0315 (4)	0.0453 (11)	
C2	0.0166 (4)	0.9110 (4)	0.1475 (3)	0.0392 (10)	
H2	−0.0025	0.9968	0.1456	0.047*	
C3	−0.0627 (5)	0.8583 (4)	0.2161 (4)	0.0459 (11)	
H3A	−0.1572	0.8630	0.1844	0.055*	
H3B	−0.0462	0.7720	0.2120	0.055*	
C4	−0.0294 (4)	0.9237 (4)	0.3364 (4)	0.0438 (11)	
C5	0.2340 (4)	0.9935 (4)	0.2540 (4)	0.0384 (10)	
H5	0.1917	1.0660	0.2675	0.046*	
C6	0.3739 (4)	0.9979 (4)	0.3061 (3)	0.0393 (10)	
C7	0.4499 (4)	0.8934 (4)	0.2841 (3)	0.0390 (10)	
C8	0.5882 (5)	0.9024 (4)	0.3411 (4)	0.0466 (11)	
H8	0.6386	0.8353	0.3277	0.056*	
C9	0.6482 (5)	1.0049 (5)	0.4137 (4)	0.0494 (12)	
H9	0.7383	1.0060	0.4487	0.059*	
C10	0.5767 (5)	1.1125 (4)	0.4385 (4)	0.0447 (11)	
C11	0.4386 (5)	1.1087 (4)	0.3848 (4)	0.0431 (11)	
C12	0.3706 (5)	1.2158 (4)	0.4134 (4)	0.0549 (13)	
H12	0.2802	1.2173	0.3804	0.066*	
C13	0.4364 (6)	1.3169 (5)	0.4890 (4)	0.0613 (14)	
H13	0.3892	1.3854	0.5061	0.074*	
C14	0.5722 (6)	1.3197 (5)	0.5408 (4)	0.0623 (14)	
H14	0.6152	1.3894	0.5910	0.075*	
C15	0.6402 (5)	1.2186 (5)	0.5165 (4)	0.0546 (13)	
H15	0.7301	1.2190	0.5517	0.065*	
C16	0.2636 (5)	0.4573 (4)	0.0400 (4)	0.0464 (11)	
H16	0.2234	0.4435	0.0940	0.056*	
C17	0.3071 (5)	0.3567 (4)	−0.0233 (4)	0.0538 (13)	
H17	0.2967	0.2778	−0.0109	0.065*	
C18	0.3653 (5)	0.3757 (5)	−0.1039 (4)	0.0540 (13)	
H18	0.3962	0.3096	−0.1453	0.065*	
C19	0.3785 (4)	0.4939 (4)	−0.1242 (4)	0.0472 (12)	
C20	0.3325 (4)	0.5912 (4)	−0.0556 (4)	0.0416 (11)	
C21	0.3453 (4)	0.7150 (4)	−0.0716 (4)	0.0424 (11)	
C22	0.4021 (5)	0.7404 (5)	−0.1544 (4)	0.0532 (12)	
C23	0.4084 (5)	0.8629 (5)	−0.1670 (4)	0.0618 (14)	
H23	0.4442	0.8831	−0.2212	0.074*	
C24	0.3612 (5)	0.9528 (5)	−0.0986 (4)	0.0632 (14)	
H24	0.3659	1.0345	−0.1055	0.076*	
C25	0.3058 (5)	0.9198 (4)	−0.0182 (4)	0.0516 (12)	
H25	0.2725	0.9811	0.0264	0.062*	
C26	0.4361 (5)	0.5229 (5)	−0.2071 (4)	0.0609 (14)	
H26	0.4671	0.4599	−0.2518	0.073*	
C27	0.4466 (5)	0.6385 (6)	−0.2220 (4)	0.0629 (15)	
H27	0.4836	0.6534	−0.2775	0.076*	
C28	0.0719 (10)	0.4856 (9)	0.7005 (9)	0.078 (3)	0.732 (13)
H28	0.0342	0.4672	0.7543	0.094*	0.732 (13)
C29	0.1211 (12)	0.6333 (11)	0.5983 (8)	0.102 (4)	0.732 (13)
H29A	0.1869	0.5782	0.5812	0.153*	0.732 (13)
H29B	0.1620	0.7155	0.6253	0.153*	0.732 (13)
H29C	0.0508	0.6313	0.5336	0.153*	0.732 (13)
C30	0.0016 (13)	0.6932 (11)	0.7444 (10)	0.116 (5)	0.732 (13)
H30A	−0.0315	0.6592	0.7965	0.174*	0.732 (13)
H30B	−0.0711	0.7220	0.6956	0.174*	0.732 (13)
H30C	0.0659	0.7608	0.7822	0.174*	0.732 (13)
C28'	0.140 (3)	0.508 (2)	0.619 (2)	0.085 (9)	0.268 (13)
H28'	0.1863	0.5244	0.5694	0.102*	0.268 (13)
C29'	−0.006 (3)	0.544 (3)	0.737 (3)	0.103 (11)	0.268 (13)
H29D	−0.0919	0.5131	0.6904	0.155*	0.268 (13)
H29E	−0.0175	0.6058	0.7977	0.155*	0.268 (13)
H29F	0.0401	0.4783	0.7646	0.155*	0.268 (13)
C30'	0.058 (3)	0.710 (3)	0.657 (3)	0.107 (11)	0.268 (13)
H30D	0.0192	0.7028	0.5802	0.161*	0.268 (13)
H30E	0.1457	0.7508	0.6764	0.161*	0.268 (13)
H30F	0.0031	0.7568	0.6977	0.161*	0.268 (13)

### Atomic displacement parameters (Å^2^)

**Table:**

	*U*^11^	*U*^22^	*U*^33^	*U*^12^	*U*^13^	*U*^23^
V1	0.0477 (5)	0.0351 (5)	0.0436 (5)	0.0085 (3)	0.0187 (4)	0.0015 (3)
N1	0.077 (3)	0.040 (2)	0.047 (2)	0.008 (2)	0.021 (2)	0.002 (2)
N2	0.037 (2)	0.040 (2)	0.0375 (19)	0.0059 (16)	0.0129 (16)	0.0021 (17)
N3	0.038 (2)	0.037 (2)	0.046 (2)	0.0058 (15)	0.0151 (17)	0.0051 (17)
N4	0.046 (2)	0.040 (2)	0.046 (2)	0.0086 (16)	0.0131 (18)	0.0085 (18)
N5	0.083 (4)	0.059 (3)	0.072 (3)	0.007 (3)	0.015 (3)	0.017 (3)
O1	0.046 (2)	0.0448 (18)	0.0514 (18)	0.0068 (14)	0.0150 (15)	−0.0074 (16)
O2	0.051 (2)	0.066 (2)	0.0485 (19)	0.0130 (17)	0.0027 (17)	0.0049 (18)
O3	0.085 (3)	0.042 (2)	0.054 (2)	0.0017 (17)	0.0326 (19)	−0.0018 (17)
O4	0.0460 (19)	0.0415 (18)	0.0442 (17)	0.0115 (14)	0.0158 (15)	−0.0021 (15)
O5	0.066 (2)	0.0460 (19)	0.056 (2)	0.0083 (15)	0.0253 (18)	0.0080 (16)
O6	0.129 (5)	0.050 (3)	0.145 (5)	0.005 (3)	−0.002 (4)	0.009 (3)
C1	0.050 (3)	0.042 (3)	0.046 (3)	0.002 (2)	0.018 (2)	0.006 (2)
C2	0.037 (3)	0.038 (2)	0.043 (2)	0.0082 (18)	0.013 (2)	0.004 (2)
C3	0.047 (3)	0.046 (3)	0.046 (3)	0.006 (2)	0.019 (2)	0.003 (2)
C4	0.044 (3)	0.045 (3)	0.042 (3)	0.010 (2)	0.018 (2)	0.000 (2)
C5	0.044 (3)	0.033 (2)	0.045 (2)	0.0075 (19)	0.022 (2)	0.009 (2)
C6	0.042 (3)	0.040 (2)	0.039 (2)	0.0059 (19)	0.015 (2)	0.011 (2)
C7	0.042 (3)	0.044 (3)	0.036 (2)	0.0063 (19)	0.017 (2)	0.010 (2)
C8	0.046 (3)	0.051 (3)	0.045 (3)	0.012 (2)	0.016 (2)	0.009 (2)
C9	0.045 (3)	0.057 (3)	0.048 (3)	0.010 (2)	0.015 (2)	0.014 (3)
C10	0.046 (3)	0.049 (3)	0.042 (2)	−0.002 (2)	0.016 (2)	0.011 (2)
C11	0.048 (3)	0.041 (3)	0.043 (2)	0.003 (2)	0.018 (2)	0.006 (2)
C12	0.058 (3)	0.045 (3)	0.054 (3)	0.004 (2)	0.009 (3)	−0.002 (2)
C13	0.074 (4)	0.039 (3)	0.065 (3)	0.001 (2)	0.017 (3)	−0.002 (3)
C14	0.071 (4)	0.051 (3)	0.057 (3)	−0.013 (3)	0.013 (3)	−0.004 (3)
C15	0.054 (3)	0.055 (3)	0.051 (3)	−0.009 (2)	0.012 (2)	0.005 (3)
C16	0.046 (3)	0.034 (2)	0.058 (3)	0.0005 (19)	0.015 (2)	0.003 (2)
C17	0.048 (3)	0.038 (3)	0.065 (3)	0.001 (2)	0.007 (3)	−0.004 (2)
C18	0.049 (3)	0.049 (3)	0.053 (3)	0.010 (2)	0.009 (2)	−0.013 (2)
C19	0.040 (3)	0.052 (3)	0.042 (3)	0.008 (2)	0.008 (2)	−0.005 (2)
C20	0.039 (3)	0.044 (3)	0.039 (2)	0.008 (2)	0.010 (2)	0.002 (2)
C21	0.040 (3)	0.048 (3)	0.038 (2)	0.008 (2)	0.008 (2)	0.008 (2)
C22	0.051 (3)	0.066 (3)	0.045 (3)	0.009 (2)	0.015 (2)	0.012 (3)
C23	0.068 (4)	0.075 (4)	0.053 (3)	0.005 (3)	0.024 (3)	0.027 (3)
C24	0.073 (4)	0.059 (3)	0.067 (3)	0.010 (3)	0.023 (3)	0.028 (3)
C25	0.055 (3)	0.044 (3)	0.058 (3)	0.008 (2)	0.014 (3)	0.013 (2)
C26	0.056 (3)	0.073 (4)	0.048 (3)	0.015 (3)	0.017 (3)	−0.005 (3)
C27	0.064 (4)	0.083 (4)	0.045 (3)	0.011 (3)	0.025 (3)	0.006 (3)
C28	0.075 (7)	0.063 (6)	0.091 (7)	−0.004 (5)	0.009 (5)	0.022 (6)
C29	0.131 (10)	0.096 (9)	0.083 (7)	0.000 (7)	0.017 (7)	0.043 (7)
C30	0.141 (10)	0.096 (8)	0.099 (8)	0.062 (7)	0.021 (7)	0.001 (7)
C28'	0.10 (2)	0.065 (17)	0.095 (19)	0.000 (13)	0.022 (16)	0.020 (15)
C29'	0.11 (3)	0.10 (2)	0.11 (2)	0.001 (19)	0.04 (2)	0.017 (19)
C30'	0.11 (2)	0.09 (2)	0.12 (3)	0.012 (17)	0.02 (2)	0.02 (2)

### Geometric parameters (Å, °)

**Table:**

V1—O5	1.587 (3)	C10—C11	1.424 (6)
V1—O4	1.975 (3)	C11—C12	1.424 (7)
V1—O1	2.004 (3)	C12—C13	1.375 (7)
V1—N2	2.057 (3)	C12—H12	0.9300
V1—N3	2.168 (3)	C13—C14	1.398 (7)
V1—N4	2.366 (4)	C13—H13	0.9300
N1—C4	1.326 (6)	C14—C15	1.361 (7)
N1—H1A	0.8600	C14—H14	0.9300
N1—H1B	0.8600	C15—H15	0.9300
N2—C5	1.284 (5)	C16—C17	1.399 (6)
N2—C2	1.480 (5)	C16—H16	0.9300
N3—C16	1.346 (5)	C17—C18	1.370 (7)
N3—C20	1.359 (5)	C17—H17	0.9300
N4—C25	1.329 (6)	C18—C19	1.398 (7)
N4—C21	1.376 (6)	C18—H18	0.9300
N5—C28	1.299 (10)	C19—C20	1.423 (6)
N5—C30'	1.36 (3)	C19—C26	1.432 (7)
N5—C29'	1.36 (3)	C20—C21	1.438 (6)
N5—C29	1.439 (11)	C21—C22	1.410 (6)
N5—C28'	1.48 (3)	C22—C23	1.403 (7)
N5—C30	1.503 (11)	C22—C27	1.443 (7)
O1—C1	1.297 (5)	C23—C24	1.377 (7)
O2—C1	1.226 (6)	C23—H23	0.9300
O3—C4	1.228 (5)	C24—C25	1.406 (7)
O4—C7	1.310 (5)	C24—H24	0.9300
O6—C28	1.290 (12)	C25—H25	0.9300
O6—C28'	1.31 (3)	C26—C27	1.342 (8)
C1—C2	1.547 (6)	C26—H26	0.9300
C2—C3	1.531 (6)	C27—H27	0.9300
C2—H2	0.9800	C28—H28	0.9300
C3—C4	1.530 (6)	C29—H29A	0.9600
C3—H3A	0.9700	C29—H29B	0.9600
C3—H3B	0.9700	C29—H29C	0.9600
C5—C6	1.436 (6)	C30—H30A	0.9600
C5—H5	0.9300	C30—H30B	0.9600
C6—C7	1.435 (6)	C30—H30C	0.9600
C6—C11	1.462 (6)	C28'—H28'	0.9300
C7—C8	1.432 (6)	C29'—H29D	0.9600
C8—C9	1.352 (6)	C29'—H29E	0.9600
C8—H8	0.9300	C29'—H29F	0.9600
C9—C10	1.440 (7)	C30'—H30D	0.9600
C9—H9	0.9300	C30'—H30E	0.9600
C10—C15	1.419 (6)	C30'—H30F	0.9600
			
O5—V1—O4	101.87 (16)	C15—C10—C11	120.5 (4)
O5—V1—O1	103.57 (16)	C15—C10—C9	121.3 (5)
O4—V1—O1	153.16 (14)	C11—C10—C9	118.2 (4)
O5—V1—N2	103.66 (15)	C10—C11—C12	116.6 (4)
O4—V1—N2	86.43 (13)	C10—C11—C6	119.8 (4)
O1—V1—N2	79.44 (13)	C12—C11—C6	123.6 (4)
O5—V1—N3	91.99 (15)	C13—C12—C11	121.0 (5)
O4—V1—N3	95.45 (12)	C13—C12—H12	119.5
O1—V1—N3	92.06 (13)	C11—C12—H12	119.5
N2—V1—N3	163.53 (14)	C12—C13—C14	121.9 (5)
O5—V1—N4	164.43 (15)	C12—C13—H13	119.1
O4—V1—N4	79.26 (13)	C14—C13—H13	119.1
O1—V1—N4	78.54 (13)	C15—C14—C13	118.8 (5)
N2—V1—N4	91.91 (13)	C15—C14—H14	120.6
N3—V1—N4	72.46 (13)	C13—C14—H14	120.6
C4—N1—H1A	120.0	C14—C15—C10	121.3 (5)
C4—N1—H1B	120.0	C14—C15—H15	119.4
H1A—N1—H1B	120.0	C10—C15—H15	119.4
C5—N2—C2	119.5 (4)	N3—C16—C17	122.6 (4)
C5—N2—V1	128.8 (3)	N3—C16—H16	118.7
C2—N2—V1	111.7 (2)	C17—C16—H16	118.7
C16—N3—C20	117.8 (4)	C18—C17—C16	119.3 (5)
C16—N3—V1	122.2 (3)	C18—C17—H17	120.4
C20—N3—V1	120.0 (3)	C16—C17—H17	120.4
C25—N4—C21	117.7 (4)	C17—C18—C19	120.5 (4)
C25—N4—V1	129.1 (3)	C17—C18—H18	119.8
C21—N4—V1	113.2 (3)	C19—C18—H18	119.8
C28—N5—C30'	178.4 (15)	C18—C19—C20	116.8 (4)
C28—N5—C29'	52.5 (14)	C18—C19—C26	124.7 (4)
C30'—N5—C29'	127 (2)	C20—C19—C26	118.6 (5)
C28—N5—C29	123.9 (9)	N3—C20—C19	123.1 (4)
C30'—N5—C29	56.0 (15)	N3—C20—C21	117.7 (4)
C29'—N5—C29	166.6 (15)	C19—C20—C21	119.2 (4)
C28—N5—C28'	63.0 (11)	N4—C21—C22	122.7 (4)
C30'—N5—C28'	117.0 (19)	N4—C21—C20	116.5 (4)
C29'—N5—C28'	114.5 (18)	C22—C21—C20	120.8 (4)
C29—N5—C28'	61.0 (11)	C23—C22—C21	117.7 (5)
C28—N5—C30	120.2 (9)	C23—C22—C27	124.4 (5)
C30'—N5—C30	59.9 (16)	C21—C22—C27	117.9 (5)
C29'—N5—C30	69.4 (15)	C24—C23—C22	119.6 (5)
C29—N5—C30	115.9 (9)	C24—C23—H23	120.2
C28'—N5—C30	175.2 (12)	C22—C23—H23	120.2
C1—O1—V1	119.2 (3)	C23—C24—C25	119.2 (5)
C7—O4—V1	132.7 (3)	C23—C24—H24	120.4
C28—O6—C28'	68.3 (13)	C25—C24—H24	120.4
O2—C1—O1	125.4 (4)	N4—C25—C24	123.1 (5)
O2—C1—C2	120.4 (4)	N4—C25—H25	118.4
O1—C1—C2	114.2 (4)	C24—C25—H25	118.4
N2—C2—C3	110.1 (4)	C27—C26—C19	121.8 (5)
N2—C2—C1	107.0 (3)	C27—C26—H26	119.1
C3—C2—C1	106.6 (3)	C19—C26—H26	119.1
N2—C2—H2	111.0	C26—C27—C22	121.7 (5)
C3—C2—H2	111.0	C26—C27—H27	119.1
C1—C2—H2	111.0	C22—C27—H27	119.1
C4—C3—C2	114.5 (4)	O6—C28—N5	120.8 (9)
C4—C3—H3A	108.6	O6—C28—H28	119.6
C2—C3—H3A	108.6	N5—C28—H28	119.6
C4—C3—H3B	108.6	N5—C29—H29A	109.5
C2—C3—H3B	108.6	N5—C29—H29B	109.5
H3A—C3—H3B	107.6	N5—C29—H29C	109.5
O3—C4—N1	123.5 (4)	N5—C30—H30A	109.5
O3—C4—C3	121.1 (4)	N5—C30—H30B	109.5
N1—C4—C3	115.4 (4)	N5—C30—H30C	109.5
N2—C5—C6	126.9 (4)	O6—C28'—N5	107.7 (19)
N2—C5—H5	116.6	O6—C28'—H28'	126.1
C6—C5—H5	116.6	N5—C28'—H28'	126.1
C7—C6—C5	120.7 (4)	N5—C29'—H29D	109.5
C7—C6—C11	119.7 (4)	N5—C29'—H29E	109.5
C5—C6—C11	119.6 (4)	H29D—C29'—H29E	109.5
O4—C7—C8	118.0 (4)	N5—C29'—H29F	109.5
O4—C7—C6	124.1 (4)	H29D—C29'—H29F	109.5
C8—C7—C6	117.9 (4)	H29E—C29'—H29F	109.5
C9—C8—C7	122.5 (4)	N5—C30'—H30D	109.5
C9—C8—H8	118.8	N5—C30'—H30E	109.5
C7—C8—H8	118.8	H30D—C30'—H30E	109.5
C8—C9—C10	121.9 (5)	N5—C30'—H30F	109.5
C8—C9—H9	119.1	H30D—C30'—H30F	109.5
C10—C9—H9	119.1	H30E—C30'—H30F	109.5
			
O5—V1—N2—C5	102.7 (4)	C7—C8—C9—C10	−0.1 (7)
O4—V1—N2—C5	1.4 (4)	C8—C9—C10—C15	178.7 (4)
O1—V1—N2—C5	−155.7 (4)	C8—C9—C10—C11	0.6 (7)
N3—V1—N2—C5	−95.8 (6)	C15—C10—C11—C12	0.4 (6)
N4—V1—N2—C5	−77.7 (4)	C9—C10—C11—C12	178.5 (4)
O5—V1—N2—C2	−80.4 (3)	C15—C10—C11—C6	−178.9 (4)
O4—V1—N2—C2	178.3 (3)	C9—C10—C11—C6	−0.7 (6)
O1—V1—N2—C2	21.2 (3)	C7—C6—C11—C10	0.4 (6)
N3—V1—N2—C2	81.1 (5)	C5—C6—C11—C10	178.7 (4)
N4—V1—N2—C2	99.2 (3)	C7—C6—C11—C12	−178.7 (4)
O5—V1—N3—C16	2.5 (4)	C5—C6—C11—C12	−0.5 (6)
O4—V1—N3—C16	104.7 (3)	C10—C11—C12—C13	0.1 (7)
O1—V1—N3—C16	−101.1 (4)	C6—C11—C12—C13	179.3 (4)
N2—V1—N3—C16	−159.5 (4)	C11—C12—C13—C14	0.1 (8)
N4—V1—N3—C16	−178.4 (4)	C12—C13—C14—C15	−0.8 (8)
O5—V1—N3—C20	−176.8 (3)	C13—C14—C15—C10	1.3 (7)
O4—V1—N3—C20	−74.7 (3)	C11—C10—C15—C14	−1.1 (7)
O1—V1—N3—C20	79.5 (3)	C9—C10—C15—C14	−179.2 (5)
N2—V1—N3—C20	21.2 (7)	C20—N3—C16—C17	2.2 (7)
N4—V1—N3—C20	2.2 (3)	V1—N3—C16—C17	−177.2 (3)
O5—V1—N4—C25	−178.2 (6)	N3—C16—C17—C18	−0.7 (7)
O4—V1—N4—C25	−82.5 (4)	C16—C17—C18—C19	−1.4 (7)
O1—V1—N4—C25	82.3 (4)	C17—C18—C19—C20	1.7 (7)
N2—V1—N4—C25	3.5 (4)	C17—C18—C19—C26	−179.2 (4)
N3—V1—N4—C25	178.2 (4)	C16—N3—C20—C19	−1.8 (6)
O5—V1—N4—C21	1.1 (8)	V1—N3—C20—C19	177.6 (3)
O4—V1—N4—C21	96.8 (3)	C16—N3—C20—C21	178.9 (4)
O1—V1—N4—C21	−98.4 (3)	V1—N3—C20—C21	−1.7 (5)
N2—V1—N4—C21	−177.2 (3)	C18—C19—C20—N3	−0.1 (7)
N3—V1—N4—C21	−2.5 (3)	C26—C19—C20—N3	−179.3 (4)
O5—V1—O1—C1	96.2 (3)	C18—C19—C20—C21	179.1 (4)
O4—V1—O1—C1	−64.9 (4)	C26—C19—C20—C21	0.0 (7)
N2—V1—O1—C1	−5.5 (3)	C25—N4—C21—C22	1.6 (6)
N3—V1—O1—C1	−171.3 (3)	V1—N4—C21—C22	−177.8 (3)
N4—V1—O1—C1	−99.6 (3)	C25—N4—C21—C20	−178.0 (4)
O5—V1—O4—C7	−97.9 (4)	V1—N4—C21—C20	2.6 (5)
O1—V1—O4—C7	63.3 (5)	N3—C20—C21—N4	−0.8 (6)
N2—V1—O4—C7	5.3 (4)	C19—C20—C21—N4	179.9 (4)
N3—V1—O4—C7	168.9 (4)	N3—C20—C21—C22	179.6 (4)
N4—V1—O4—C7	98.0 (4)	C19—C20—C21—C22	0.3 (7)
V1—O1—C1—O2	171.5 (4)	N4—C21—C22—C23	−1.1 (7)
V1—O1—C1—C2	−11.2 (5)	C20—C21—C22—C23	178.5 (4)
C5—N2—C2—C3	−98.3 (4)	N4—C21—C22—C27	−179.6 (4)
V1—N2—C2—C3	84.5 (3)	C20—C21—C22—C27	0.0 (7)
C5—N2—C2—C1	146.2 (4)	C21—C22—C23—C24	0.7 (8)
V1—N2—C2—C1	−31.0 (4)	C27—C22—C23—C24	179.1 (5)
O2—C1—C2—N2	−155.0 (4)	C22—C23—C24—C25	−0.9 (8)
O1—C1—C2—N2	27.5 (5)	C21—N4—C25—C24	−1.7 (7)
O2—C1—C2—C3	87.2 (5)	V1—N4—C25—C24	177.6 (4)
O1—C1—C2—C3	−90.3 (4)	C23—C24—C25—N4	1.4 (8)
N2—C2—C3—C4	61.0 (5)	C18—C19—C26—C27	−179.6 (5)
C1—C2—C3—C4	176.7 (4)	C20—C19—C26—C27	−0.5 (7)
C2—C3—C4—O3	33.3 (6)	C19—C26—C27—C22	0.9 (8)
C2—C3—C4—N1	−147.8 (4)	C23—C22—C27—C26	−178.9 (5)
C2—N2—C5—C6	177.5 (4)	C21—C22—C27—C26	−0.6 (8)
V1—N2—C5—C6	−5.9 (6)	C28'—O6—C28—N5	3.5 (14)
N2—C5—C6—C7	4.5 (6)	C29'—N5—C28—O6	165 (2)
N2—C5—C6—C11	−173.8 (4)	C29—N5—C28—O6	0.5 (13)
V1—O4—C7—C8	172.9 (3)	C28'—N5—C28—O6	−3.2 (13)
V1—O4—C7—C6	−7.7 (6)	C30—N5—C28—O6	−179.2 (8)
C5—C6—C7—O4	2.3 (6)	C28—O6—C28'—N5	−2.8 (11)
C11—C6—C7—O4	−179.4 (4)	C28—N5—C28'—O6	2.9 (11)
C5—C6—C7—C8	−178.3 (4)	C30'—N5—C28'—O6	−175.3 (18)
C11—C6—C7—C8	0.0 (6)	C29'—N5—C28'—O6	−8(2)
O4—C7—C8—C9	179.3 (4)	C29—N5—C28'—O6	−174 (2)
C6—C7—C8—C9	−0.1 (6)		

### Hydrogen-bond geometry (Å, °)

**Table:**

*D*—H···*A*	*D*—H	H···*A*	*D*···*A*	*D*—H···*A*
N1—H1A···O3^i^	0.86	2.05	2.909 (5)	175
N1—H1B···O6^ii^	0.86	2.00	2.852 (6)	169
C16—H16···O5	0.93	2.52	2.975 (5)	111
C29—H29A···O6	0.96	2.41	2.764 (11)	102
C29—H29C···O6^ii^	0.96	2.56	3.511 (12)	173
C25—H25···O2^iii^	0.93	2.46	3.275 (6)	147
C17—H17···O2^iv^	0.93	2.54	3.288 (6)	138

Symmetry codes: (i) −*x*, −*y*+2, −*z*+1; (ii) −*x*, −*y*+1, −*z*+1; (iii) −*x*, −*y*+2, −*z*; (iv) −*x*, −*y*+1, −*z*.

## References

[bb1] Bruker (1996). *SMART* and *SAINT* Bruker AXS, Inc., Madison, Wisconsin, USA.

[bb2] Diego, D. R., Agustin, G., Ramon, V., Carlo, M., Andrea, I. & Dante, M. (2003). *Dalton Trans.* pp. 1813–1820.

[bb3] Hoshina, G., Tsuchimoto, M., Ohba, S., Nakajima, K., Uekusa, H., Ohashi, Y., Ishida, H. & Kojima, M. (1998). *Inorg. Chem.* **37**, 142–145. 10.1021/ic9705958 11670274

[bb4] Kenji, K., Makoto, T., Ken, H., Naohisa, Y. & Yoshitane, K. (2000). *Inorg. Chim. Acta*, **305**, 172–183.

[bb5] Otieno, T., Bond, M. R., Mokry, L. M., Walter, R. B. & Carrano, C. J. (1996). *J. Chem. Soc. Chem. Commun.* pp. 37–38.

[bb6] Sheldrick, G. M. (1996). *SADABS* University of Göttingen, Germany.

[bb7] Sheldrick, G. M. (2008). *Acta Cryst.* A**64**, 112–122. 10.1107/S0108767307043930 18156677

[bb8] Thompson, K. H. & Orvig, C. (2006). *Dalton Trans.* pp. 761–764. 10.1039/b513476e 16437168

